# Platinum and Taxane Based Adjuvant and Neoadjuvant Chemotherapy in Early Triple-Negative Breast Cancer: A Narrative Review

**DOI:** 10.3389/fphar.2021.770663

**Published:** 2021-12-06

**Authors:** Hao Tian, Dandan Ma, Xuanni Tan, Wenting Yan, Xiujuan Wu, Cheng He, Ling Zhong, Yan Zhang, Bingjie Yu, Yi Zhang, Xiaowei Qi

**Affiliations:** ^1^ Department of Breast and Thyroid Surgery, Southwest Hospital, Third Military Medical University (Army Medical University), Shapingba, China; ^2^ Department of Medical Oncology, Sanofi China Corporate, Shanghai, China

**Keywords:** adjuvant chemotherapy, neoadjuvant therapy, platinum, taxane, triple negative breast cancer

## Abstract

Platinum (Pt) derivatives such as cisplatin and carboplatin are the class of drugs with proven activity against triple-negative breast cancer (TNBC). This is due to the ability of Pt compounds to interfere with the DNA repair mechanisms of the neoplastic cells. Taxanes have been efficacious against estrogen receptor-negative tumors and act by disruption of microtubule function. Due to their distinct mechanisms of action and routes of metabolism, the combination of the Pt agents and taxanes results in reduced systemic toxicity, which is ideal for treating TNBC. Also, the sensitivity of *BRCA1*-mutated cells to taxanes remains unsolved as *in vitro* evidence indicates resistance against taxanes due to *BRCA1* mutations. Recent evidence suggests that the combination of carboplatin and paclitaxel resulted in better pathological complete response (pCR) in patients with TNBC, both in neoadjuvant and adjuvant settings. *In vitro* studies showed sequential dependency and optimal time scheduling of Pt- and taxane-based chemotherapy. Also, combining carboplatin with docetaxel in the NAC regimen yields an excellent pCR in patients with *BRCA*-associated and wild-type TNBC. TNBC is a therapeutic challenge that can be tackled by identifying new therapeutic sub-targets and specific cross-sections that can be benefitted from the addition of Pt- and taxane-based chemotherapy. This review summarizes the merits as well as the mechanism of Pt- and taxane-based adjuvant and neoadjuvant chemotherapies in early TNBC from the available and ongoing clinical studies.

## Introduction

Triple-negative breast cancer (TNBC) is a subtype of breast cancer (BC) that collectively represents 15–20% of all the BC reported (Dent et al., 2007). It is usually associated with rapid disease progression, higher mortality rate, poorer prognosis, and distant recurrences when compared to other forms of breast cancer. Despite having larger tumors and a marked rate of node positivity, patients in the triple-negative category exhibit a weaker relationship between tumor size and node status (Dent et al., 2007).

In the TNBC subtype, negative expressions of progesterone receptor (PR), estrogen receptor (ER), and human epidermal growth factor receptor-2 (HER2) are observed (Jhan and Andrechek, 2017). TNBC is a heterogeneous disease that is classified based on the specific histological characteristics of the tumor and the expression of single molecular markers (BCL2, p53, MDR-1, Ki67, etc.) and manifests into a range of clinical outcomes (Jhan and Andrechek, 2017; Diana et al., 2020).

The current treatment approach for TNBC consists of chemotherapy drugs such as anthracyclines, taxanes, platinum (Pt) derivatives, and targeted therapies such as angiogenesis inhibitors (bevacizumab), PARP1 inhibitors, EGFR inhibitors, tyrosine kinase and ERK inhibitors, and mTOR inhibitors (Mustacchi and De Laurentiis, 2015). Anthracyclines and taxanes have proven efficacy in both early-stage and metastatic ER-negative BC tumors and hence, both the classes are designated as first-line treatment of TNBC (Mustacchi and De Laurentiis, 2015).

*BRCA1* and *BRCA2* genes synthesize proteins that aid the repair of damaged DNA. They are also called tumor suppressor genes as they regulate cell division and are susceptible to the development of pathogenic mutations which can subsequently lead to carcinogenesis (Filippini and Vega, 2013; Diana et al., 2020). About 71% of germline *BRCA1* mutation carriers and 25% of germline *BRCA2* mutation carriers are affected by TNBC phenotype (Peshkin et al., 2010). Recently, Pt derivatives such as cisplatin and carboplatin have shown a revived interest in the treatment of TNBC. Preclinical data also suggest a favorable activity of Pt agents in TNBC and *BRCA1*-associated breast cancer (Rapoport et al., 2014).

The purpose of this narrative review is to objectively summarize the efficacy of Pt- and taxane-based neoadjuvant chemotherapy (NAC) and adjuvant chemotherapy in patients with early TNBC, as well as analyze its underlying pharmacological mechanism from a broader clinical perspective.

## Methodology

A literature search was performed on PubMed for articles published in English from inception till May 2021, focusing on MeSH terms ‘triple-negative breast cancer’, ‘taxanes’, and ‘platinum agents’ in the context of ‘adjuvant’ and ‘neoadjuvant’ settings. The same search terms were used for the Embase and ClinicalTrials.gov registry of clinical trials. Abstracts from the annual meetings for the American Society of Clinical Oncology (ASCO) and European Society for Medical Oncology (ESMO) from 2015 to 2021 were also screened. We excluded publications if no clinical comparative information about the pCR or survival outcomes were reported (Supplementary Figure S1). A brief summary of the studies included in this review has been shown in Tables 1 and 2.

**TABLE 1:** Summary of neoadjuvant studies with Pt derivatives and taxane combinations.

Study	Phase	Trial number	Molecular subtype of breast cancer	Intervention	Comparator	Outcomes
von Minckwitz et al. (2014)	II	NCT01426880 (Geparsixto)	TNBC	P + A Bev	P + A Bev with Cb	pCR: 43.7 vs. 36.9%, (OR = 1.33, 95% CI 0.96–1.85; *p =* 0.107)
						TNBC: pCR: 53.2 vs. 36.9% (*p =* 0.005)
						Grade 3 or 4 neutropenia (192 [65%] vs. 79 [27%])
						Grade 3 or 4 anemia (45 [15%] vs. 1 [<1%])
						Grade 3 or 4 thrombocytopenia (42 [14%] vs. 1 [<1%])
						Grade 3 or 4 diarrhea (51 [17%] vs. 32 [11%])
						Dose discontinuations (141 [48%] vs. 114 [39%]) (*p =* 0.031).
						Frequency of grade 3 or 4 hematological events decreased from 82% (n = 135) to 70% (n = 92) and grade 3 or 4 non-hematological events from 78% (n = 128)
						to 59% (n = 77) in the Cb arm when the dose of Cb was reduced from AUC 2.0
						to 1.5.
Loibl et al. (2018)	III	NCT02032277 (BrighTNess)	TNBC	Veli + P + Cb → AC	P → AC and P + Cb → AC	pCR: Veli + P + Cb vs. P alone (168 [53%] vs. 49 [31%]), (*p* < 0.0001),
						pCR: Veli + P + Cb b vs. P + Cb (168 [53%] vs. 92 [58%]), *p =* 0.36).
Wu et al. (2018), Sharma et al. (2018)	II	ChiCTR-TRC-14005019	TNBC	TEL	TE	TpCR: TEL vs. TE (38.7% [24/62] vs. 12.7% [8/63]), (OR: 4.342, 95% CI 1.764–10.687; *p* = 0.001)
						ORR: TEL vs. TE (93.5% [58/62] vs. 73.0% [46/63])
						Grade 3–4 anemia and thrombocytopenia: TEL vs. TE (52.5 vs. 10.0% and 34.4 vs. 1.7% respectively)
Gluz et al. (2018)	II	NCT01815242 (WSG-ADAPT-TN)	TNBC	Gem + nab-P	Cb + nab-P	pCR for 3-years EFS: 92 vs. 71%), *p* < 0.001)
						pCR for 3-years OS: 99.1 vs. 81.6%), *p* < 0.001)
						3-years EFS: 77.6 vs. 80.8%), *p =* 0.48)
						3-years OS: 84.7 vs. 92.2%), *p =* 0.08).
Sharma et al. (2021)	II	NCT02413320 (NeoSTOP)	TNBC	Cb + P → AC	Cb + T	pCR: 54%, RCB 0 + 1: 67%
						pCR in patients with BRCA TNBC: 59%
						pCR in patients with wild-type TNBC: 56%
						At least one grade 3: 21%, At least one grade 4: 7%
Zhang et al. (2020)	II	NCT03154749 (NeoCART)	TNBC	T + Cb	E + C → T	pCR: 27 (61.4%) vs. 17 (38.6%) (OR: 2.52, 95% CI 2.4–43.1; *p =* 0.033)
						stage II pCR: 73.3% (22/30) vs. 48.4% (15/31) (*p =* 0.046)
						stage III pCR: 35.7% (5/14) vs. 15.4% (2/13) (*p =* 0.384).
						Grade 3/4 AEs include anemia (4.5%), thrombocytopenia (2.3%), neutropenia (2.3%) and ALT/AST increased (2.3%) in the T + Cb group.
Sikov et al. (2015), Sikov et al. (2019)	II	NCT00861705 (CALGB 40603)	TNBC	P + Cb + Bev → AC	P + Bev → AC	pCR on addition of either: Cb (60 vs. 44%; *p* = 0.0018) or Bev (59 vs. 48%; *p =* 0.0089)
						pCR breast/axilla: Cb (54 vs. 41%; *p =* 0.0029)

A, doxorubicin; AUC, area under curve; Bev, bevacizumab; C, cyclophosphamide; CI, confidence-interval Cb, carboplatin; E, epirubicin; EFS, event-free survival; Gem, gemcitabine; nab-P, albumin paclitaxel; OR, odds Ratio; OS, overall survival, P, paclitaxel, PCR, pathological complete response; T, docetaxel; TNBC, triple negative breast cancer; Veli, veliparib; L, lobaplatin.

**TABLE 2:** Summary of adjuvant studies with Pt derivatives and taxane combinations.

Study	Phase	Trial number	Molecular subtype of breast cancer	Intervention	Comparator	Outcomes
Du et al. (2020), Burstein et al. (2019)	II	NCT01150513	TNBC	T/P + Cb	EC→T	5- year DFS: 84.4 vs. 85.8%, (*P*non-inferiority = 0.034)
						5- year OS: 93.5 vs. 94.4%, (*p* = 0.770)
						Grade 3/4 adverse events: 48.7% (75/154) vs. 68.9% (106/154)
Yu et al. (2020), Korde et al. (2021)	III	NCT01216111 (PATTERN))	TNBC	P + Cb	CEF→T	5- year DFS: 86.5 vs. 80.3%, (HR = 0.65; 95% CI, 0.44–0.96; *p =* 0.03)
						RFS: 91.2 vs. 84.4%, (HR = 0.54; 95% CI, 0.34–0.88; *p =* 0.01)
						OS: 93.4 vs. 89.8%; (HR = 0.71; 95% CI, 0.42–1.22; *p =* 0.22)
						DDFS: 92.6 vs. 87.9%; (HR = 0.59; 95% CI, 0.35–0.999; *p =* 0.05)
Wang et al. (2019)	III	NCT01378533	TNBC	P + Cb with G-CSF	EC→P with G-CSF	DFS: (HR = 0.305, 95% CI = 0.134–0.693; *p =* 0.0046)
						3-years DFS: 93.7 vs. 77.9%
						3-years OS: 98.4 vs. 92.6%, *p =* 0.0268
						Grade 3/4: 48.5 vs. 21.9%; *p =* 0.002
Nasr et al. (2015)	III		TNBC	FEC→ T	FEC→ T + Cb	mDFS:28 vs. 24 months, (*p =* 0.05)
						mOS: 37 vs. 29 months, (*p =* 0.04)
						distant metastasis recurrence rates: 26 vs. 37%
Frasci et al. (2009)	II		TNBC	Cis + E + P with G-CSF	NA	pCR: 46 women (62%; 95% confidence interval 50–73) in both breast and axilla.
						DFS: 41-months (range 3–119), 13 events (nine distant metastases) distant disease-free survival = 84%
						Five-year DFS in pCRs = 90%
						Five-year DFS in non-pCRs, = 56%.
						Severe neutropenia = 23 (31%)
						Severe anemia = 8 (10.8%)
						Severe non-hematological in <20% of patients

A, doxorubicin; C, cyclophosphamide; Cb, carboplatin; Cis, cisplatin; DDFS, Distant disease-free survival; DFS, Disease-free survival; E, epirubicin; F,5-fluorouracil; G-CSF, granulocyte stimulating factor; mDFS, median disease-free survival; mOS, median overall survival, P, paclitaxel, PCR, pathological complete response; RFS, Relapse-free survival; T, docetaxel; TNBC, Triple negative breast cancer.

## The Pharmacological Mechanism of the Combination of Taxanes and Pt Derivatives

**FIGURE 1:**
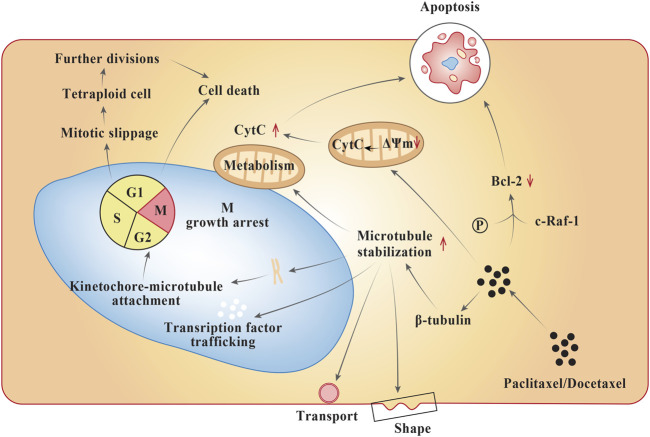
Mechanism of action of taxanes in neoplastic cells.

Although taxanes such as paclitaxel and docetaxel share a close resemblance in their molecular structure, they exhibit diverse pharmacology (Dorr, 1997). Both the taxanes bind to ß-subunit of tubulins in the neoplastic cell (Figure 1), influence microtubule polymerization, and repress the cell cycle at G2-M stage intersection. They both undergo metabolism in the liver. Furthermore, the mode of action of both the taxanes are quite similar; however, docetaxel shows a greater affinity for tubulin binding, a higher tendency towards microtubule depolymerization inhibition, stronger antitumor activity within *in vitro* and *in vivo* models, and more potent induction of BCL-2 phosphorylation leading to apoptosis (Pienta, 2001; Yiding and Zhongyi, 2021). The specific cytochrome P-450 (CYP) enzymes responsible for their hydroxylation are CYP2C8 and CYP3A4 for paclitaxel and docetaxel, respectively. The cytotoxic activity of the taxanes are discerned to increase with prolonging the duration of exposure (Dorr, 1997). At a mechanistic level, paclitaxel acts in a reversible manner to hyper stabilize the microtubules by binding to the N-terminal 31 amino acids of the ß-tubulin subunit thereby decreasing the threshold concentration of purified tubulin subunits. Also, paclitaxel has the ability to interact *in vitro* in microtubules formation at colder temperatures (4°C) and calcium concertrations (Kampan et al., 2015). As a result, the cancer cells treated with the drug are growth arrested in metaphase on bipolar spindles. Another mechanism of action of paclitaxel involves formation of tetraploid G1 cell due to improper chromosome segregation during mitosis. This results in cell death and arrest during growth phase (Weaver, 2014). Paclitaxel also activates multiple signal-transduction pathways such as toll-like receptor-4 (TLR-4) dependent pathway (either via MyD88 dependent or independent pathway), c-Jun N-terminal kinase (JNK), P38 Mitogen activated protein kinase (MAPK), nuclear factor kappa ß (NF-κß), Janus kinase- (JAK-) signal transducer and activator of transcription factor (STAT) pathway, which may be associated with proapoptotic signaling (Kampan et al., 2015). In case of docetaxel, the mode of anti-cancer activity is similar to that of paclitaxel, except that it differs structurally from the former at either the 3′ position on the side chain or the 10′ position on the baccatin ring (Montero et al., 2005).

**FIGURE 2:**
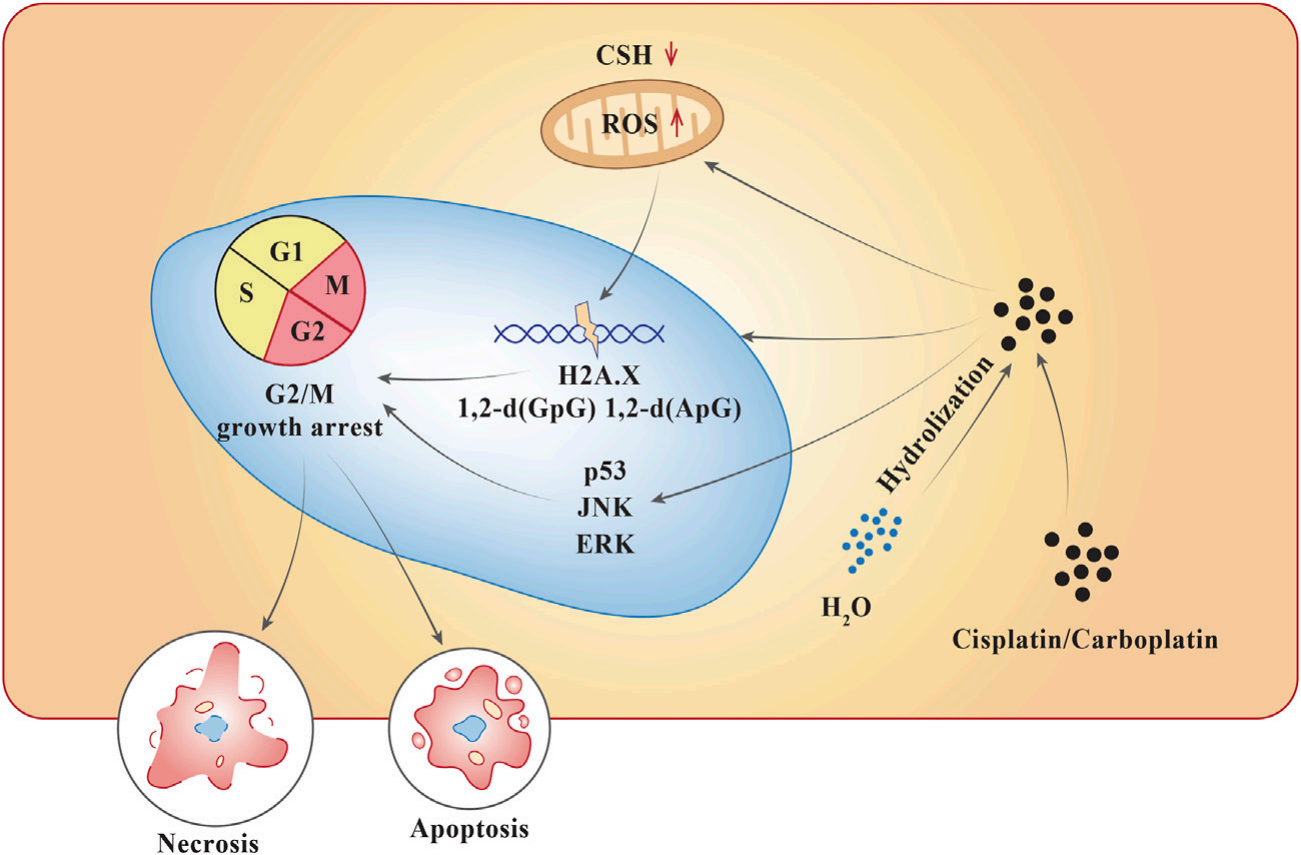
Mechanism of action of platins in neoplastic cells.

Pt compounds have a central Pt particle, surrounded by chloride (Cl^−^) molecules and ammonia groups. Pt compounds enter cells through an active carrier. Once inside the cell, the Cl^−^ particles separate, abandoning a reactive complex that interacts with the DNA (Figure 2). At a lower concentration of Cl^−^, the dissociation for Cl^−^ ions are favored, while higher intercellular concentrations of Cl^−^ generally stabilizes the drug (Bardal et al., 2011). They act by alkylating DNA purine bases, which causes guanine-guanine (GG) synthesis that leads to inter- and intra-strand cross-linkage DNA adducts, which inhibits DNA synthesis and function (Bardal et al., 2011). This interferes with DNA repair mechanisms, intrinsic mitochondrial pathway, and forms a component of endoplasmic reticulum stress, ultimately leading to either necrosis or apoptosis. Cisplatin, a Pt-based compound binds to N7 reactive center on purine bases, forming 1,2-intrastrand [d (GpG) and d (ApG)] adducts of purines, eliciting DNA injury which can lead to cell apoptosis. The pathways activated during this process include p53, extracellular-signal-regulated kinase (ERK), and c-Jun N-terminal kinase (JNK) (Dasari and Tchounwou, 2014). Similarly, carboplatin, when penetrated into the cell membrane, is subjected to hydrolysis becoming positively charged. This compound follows the same process as cisplatin and interferes with G2/M growth arrest leading to cell apoptosis or necrosis (Sousa et al., 2014). They are primarily eliminated from the circulation via renal excretion (Bardal et al., 2011; Sousa et al., 2014).

Due to their distinct mechanisms of action, Pt and taxanes are often combined in cancer therapy contributing to their synergistic action. Different routes of metabolism of these two drugs lead to reduced systemic toxicity, making it an ideal candidate for chemotherapeutic treatment among patients with BC.

The pharmacology of taxanes and Pt combination is illustrated by a well-designed study by Felici et al. utilizing a compartmental analysis for docetaxel and a non-compartmental investigation for cisplatin and 5-FU (Felici et al., 2006). They demonstrated that there was no pharmacokinetic interaction between the three drugs when given to patients with metastatic tumors while maintaining a manageable toxicity profile (Felici et al., 2006).

Multidrug resistance (MDR) is a phenomenon whereby tumor cells acquire resistance to a broad range of structurally and functionally diverse chemotherapeutic drugs, including alkylating agents, anthracyclines, vinca alkaloids, epipodophyllotoxins, and paclitaxel (Clynes, 1994). Multidrug resistance protein-1 (MRP‐1) expression in primary BC is inversely correlated with both relapse-free survival (RFS) and overall survival (OS) (Nooter et al., 1997; Filipits et al., 1999), which could be one of the possible mechanisms of action for taxane-Pt-based chemotherapy. Previously, a study demonstrated that MRP‐1 expression at diagnosis was associated with a worse prognosis in patients who received adjuvant systemic chemotherapy with cyclophosphamide, methotrexate, and 5‐fluorouracil (5-FU), in patients with small tumors (T1) and node‐negative BCs (Vulsteke et al., 2013).

*In vitro*, cells resistant to Pt compounds were found to display increased levels of MRP-1 and MRP-4 (Beretta et al., 2010). Another research reported that overexpression of MRP-1 and MRP-3 was responsible for the decrease in drug sensitivity towards vincristine, etoposide, doxorubicin, and cisplatin in patients with lung cancer (Zhang et al., 2015). Shingo Maeda et al. evaluated the antitumor effects of cisplatin and docetaxel on gastric cancer cell lines MKN-74, MKN-45, and TMK-1. Strikingly, a sequence dependency was observed in gastric cancer cells *in vitro*, since docetaxel followed by cisplatin (DC) showed a stronger antitumor effect versus cisplatin followed by docetaxel (CD) in all cell lines (survival ratios for DC vs. CD: 0.462 vs. 0.666 for MKN-45 cells; 0.691 vs. 0.838 for MKN-74 cells; 0.570 vs. 0.766 for TMK-1 cells) (Maeda et al., 2004). Also, a higher Pt accumulation (twice the Pt accumulation than control cells 1.22 ± 0.26 vs. 0.64 ± 0.03 µg/10^7^ for MKN-45 cells, 1.61 ± 0.34 vs. 0.77 ± 0.06 µg/10^7^ for MKN-74 cells, respectively, *p* < 0.05) was noted in docetaxel followed by the cisplatin group in contrast to the cells treated with only cisplatin. Combining all the study results, we hypothesis that MRP-1 upregulation is a cause for drug resistance to platinum in cancer cells, while docetaxel could suppress the MRP-1 upregulation, thus performing a synergistic effect with platinum to increase the efficacy.

Another probable mechanistic pathway for the interaction between Pt- and taxane-based chemotherapy is via MicroRNAs (miRNAs). miRNAs are small regulatory non-coding RNAs that act through multiple cellular signaling pathways by controlling the degradation and translation of their target messenger RNAs (mRNAs). miRNAs base-pair with sequences within the 3′-untranslated region (UTR), 5′-UTR, and coding sequence regions of target mRNAs (He et al., 2005).

Paclitaxel elevates the level of miR-512-3p, which induces apoptosis in carcinoma cells (Chen et al., 2010). Another study revealed that high miR-9 expression downregulates *BRCA1* activity and improves paclitaxel/taxane chemotherapy response by increasing Pt sensitivity along with longer progression-free survival (PFS) (Sun et al., 2013). miRNA let-7 binding site genetic variants located in the HIF1AN and CLDN12 genes could predict pCR to taxane- and Pt-based NAC in locally advanced BC. Polymorphisms in microRNA let-7 binding sites of the HIF1AN and CLDN12 genes can predict pCR to taxane- and Pt-based NAC in BC. (Du et al., 2019) Similarly, another study reported low *BRCA1* expression and high expression of miRNA-9 was associated with Pt sensitivity and longer PFS (Strumidło et al., 2017).

## Treatment Sequence of Taxanes and Pt-Derivatives Might Lead to Drug-Drug Interactions

Sequential treatment with taxanes and Pt derivatives might influence drug-drug interactions. Since paclitaxel undergo hepatic oxidation through the CYP system, pharmacokinetic interactions can be either sequential or schedule dependent (Scripture et al., 2005). Administration of paclitaxel following cisplatin causes an increase in myelosuppression, which is probably due to a decrease in paclitaxel clearance. In contrast, a favorable sequence-dependent pharmacodynamic interaction is seen when paclitaxel infusion is administered before carboplatin in terms of reducing platelet toxicity (Scripture et al., 2005). The systemic exposure to carboplatin alone (AUC for carboplatin = 34 μg × h/mL) showed a decrease in platelet count by 50% compared to carboplatin given after paclitaxel (AUC for carboplatin = 57 μg × h/mL), when the relationship between carboplatin and thrombocytopenia was analyzed (Scripture et al., 2005). The mechanism for this interaction remains obscure and warrants further probing. Nonetheless, the pharmacokinetic characteristics such as absorption, metabolism, distribution, and excretion of both paclitaxel and cisplatin do not vary based on their sequence of medication. A recent study suggested that sequential administration of paclitaxel 175 mg/m^2^ followed by cisplatin 75 mg/m^2^ should be preferred over a concurrent combination of both the drugs as the sequence of paclitaxel prior to cisplatin is associated with lesser toxicity (Elserafi et al., 2018). Although a similar response rate as well OS was observed in both sequential and concurrent regimens (Elserafi et al., 2018).

Some studies report that administration of paclitaxel followed by carboplatin led to a decreased formation of Pt dimers in patient’s DNA, which is speculated to contribute towards the antitumor activity of carboplatin (Baker, 1997; Calvert, 1997; Kearns and Egorin, 1997). Patients treated by paclitaxel followed by carboplatin sequence had less hematopoietic toxicity than patients treated with carboplatin followed by paclitaxel. No significant difference in the pharmacokinetics of carboplatin or paclitaxel was observed with either of the administration schedule (Baker, 1997; Calvert, 1997; Kearns and Egorin, 1997). A similar finding was observed for the combination of cisplatin and docetaxel where a significantly lower Pt DNA dimers were detected in patients treated with docetaxel followed by cisplatin (Schellens et al., 1994). While the mechanism for this interaction has not been fully understood, it is assumed that taxanes show a reduced activity when administered before Pt compounds. Thus, in most phase II/III clinical studies, the sequence of Pt agent followed by a taxane is more commonly used.

## Progress of Neoadjuvant Research on Pt Derivatives in Combination With Taxane

Apart from increasing the breast conservation rate, NAC has demonstrated comparable benefits to adjuvant chemotherapy in terms of disease-free survival (DFS) and OS (Niu et al., 2014). Of note, complete response to NAC foretells a good prognosis (Liedtke et al., 2008). Neoadjuvant therapy might have greater clinical importance for patients with TNBC than the other types of BCs (Cortazar et al., 2014). Following NAC, a higher pCR (~30–40%) is observed in TNBC than other BC subtypes (Cortazar et al., 2014). Achieving pCR had the strongest prognostic effect in patients with TNBC as it yielded better event-free survival (EFS: HR = 0.24, 95% CI 0.18–0.33) and OS (HR = 0.16, 0.11–0.25) in CTNeoBC pooled analysis compared to those without pCR. Conversely, those patients who failed to achieve pCR were at a higher risk of relapse (Kelland, 2007; Cortazar et al., 2014; Tutt et al., 2018; Dieci et al., 2019).

For many years, a taxane-anthracycline-based regimen has been incorporated as a standard for NAC regimen in treating BC. The earliest study, published in the year 2003 is the Trial B-27, drawing attention to the four cycles of sequential preoperative docetaxel to cyclophosphamide plus doxorubicin (AC) chemotherapy that provided a significantly superior outcome compared to the four cycles of AC alone (Bear et al., 2003). However, there were some flaws reported with this study design as the preoperative regimens were of different durations (4 vs. 8 cycles). Hence suggesting that the favorable results in the AC/docetaxel arm might be due to the delivery of additional cycles of chemotherapy rather than the addition of taxane. To clarify this ambiguity, 162 patients with locally advanced BC were treated with four cycles of neoadjuvant cyclophosphamide/vincristine/doxorubicin/prednisolone (CVAP). This was followed by randomization of those patients who attained complete or partial response to four additional cycles of docetaxel (100 mg/m^2^) or CVAP. Patients who completed eight cycles of chemotherapy in totality, showed a higher clinical and pathological objective response rate (ORR) with docetaxel. Moreover, patients who did not respond to four cycles CVAP, docetaxel elicited a clinical response of 67% and a pathological response of 44% (including a pCR of 15%) (Smith et al., 2002).

The addition of neoadjuvant carboplatin to taxane-anthracycline-based chemotherapy has shown potential efficacy in several recent studies conducted in TNBC patients (Table 1). In BrighTNess (Loibl et al., 2018) Trial, a phase III randomized trial, patients were given 12 doses of paclitaxel weekly plus for four cycles of carboplatin every 3 weeks plus veliparib two times a day compared to those receiving paclitaxel plus carboplatin plus veliparib placebo. The pCR achieved was quite similar in both the arms (53% in veliparib containing arm vs. 58% in veliparib placebo arm, *p* = 0.36). However, the advantage of carboplatin and paclitaxel combination over paclitaxel alone was significantly highlighted (pCR: 58% in paclitaxel plus carboplatin arm vs. 31% in paclitaxel only arm, respectively, *p* < 0.0001) in the neoadjuvant therapy of TNBC. All patients received cyclophosphamide followed by doxorubicin as the standard part of the treatment (Loibl et al., 2018).

In GeparSixto (von Minckwitz et al., 2014) randomized phase II trial, patients with TNBC received four cycles of paclitaxel 80 mg/m^2^ once a week and non-pegylated liposomal doxorubicin 20 mg/m^2^ once a week simultaneously with bevacizumab 15 mg/kg intravenously every 3 weeks. Patients were further allocated (1:1) to receive either carboplatin once a week or no carboplatin depending on their biological subtype and Ki-67 levels. This study showed that patients with additional carboplatin achieved a pCR of 53.2% which was higher than the pCR of 36.95% in the arm without carboplatin (*p =* 0.005). (von Minckwitz et al., 2014).

CALGB 40603 (Alliance), a 2 × 2 factorial, randomized phase II trial assessed the addition of carboplatin and/or bevacizumab to neoadjuvant paclitaxel once-a-week ensuing dose-dense doxorubicin and cyclophosphamide on pCR rates in stage II-III TNBC patients (Sikov et al., 2015). Earlier, CALGB 40603 showed that the addition of carboplatin to NAC significantly improved pCR (54 vs. 41%; *p =* 0.0029) in the breast/axilla compared to bevacizumab (Sikov et al., 2015; Sikov et al., 2019). However, long-term results showed no improvement in 5-years EFS with either carboplatin (HR = 0.99, 0.70–1.40) or bevacizumab (HR = 0.91, 0.64–1.29). The administration of ≥11 doses of weekly paclitaxel was linked to a better EFS (HR = 1.92, 1.33–2.77) in the exploratory analysis which was more pronounced in carboplatin-treated arms (Sikov et al., 2019).

Adding platinum to the taxane-anthracycline-based regimen can increase the AE incidence rate (Zheng et al., 2015), thus many current studies are excluding anthracyclines from NAC regimen. In a combined analysis of two cohorts by Sharma et al.,^49^carboplatin with docetaxel in NAC regimen yielded pCR in patients with *BRCA*-associated and wild-type TNBC as high as 59% (95% CI: 40–78%) and 56% (95% CI: 48–64%) respectively (*p* = 0.83) (Sharma et al., 2017). They also conducted a survival study (Sharma et al., 2018) presenting RFS and OS according to the degree of pathological response. It was seen that a pCR of 55% (100/183; 95% CI, 48–62) was obtained which is analogous to the pCR achieved when carboplatin is added to anthracycline-taxane chemotherapy. Patients with pCR had a 3-years RFS of 90% compared to the 66% in those who failed to attain pCR (HR = 0.30; 95% CI: 0.14–0.62, *p* = 0.0001). Also, the 3-years OS of 94% was noted in those with pCR while 79% in patients without pCR (HR = 0.25; 95% CI: 0.10–0.63, *p* = 0.001) (Sharma et al., 2018). Noteworthily, the pCR results observed in the aforementioned studies are remarkably higher than the traditional neoadjuvant anthracycline-taxane combinations, where at most 28–40% of TNBC patients achieve pCR (Loibl et al., 2018; von Minckwitz et al., 2014; Sharma et al., 2017; Sharma et al., 2018; Arun et al., 2011).

NeoSTOP (Neoadjuvant Study of Two Platinum Regimens in Stage I-III Triple Negative Breast Cancer) trial was aimed at assessing the efficacy of anthracycline-free and anthracycline-containing neoadjuvant carboplatin regimens in two different centers. This randomized phase II trial showed that the pCR rates (54%) were similar in both the arms, however, grade 3/4 AEs were more common in paclitaxel plus carboplatin followed by doxorubicin plus cyclophosphamide arm when compared to the carboplatin plus docetaxel only arm (73 vs. 21%, *p* < 0.0001) (Sharma et al., 2021). The NeoCART trial was a phase II, randomized, multicenter study devised to evaluate the efficacy of neoadjuvant docetaxel plus carboplatin versus epirubicin plus cyclophosphamide followed by docetaxel in early-stage TNBC patients (Zhang et al., 2020). This study reported a higher pCR in docetaxel plus carboplatin group than the standard NAC group (61.4% vs. 38·6%, OR = 2.52, 95% CI 2.4–43.1; *p =* 0.033). Noteworthily, more significantly higher pCR rates were observed in earlier disease stages and negative lymph node patients (Zhang et al., 2020). A phase II randomized study by Wu et al. also showed a pCR rate of 38.7% in the arm containing lobaplatin with epirubicin and docetaxel combination as NAC regimen compared to the pCR rate of 12.7% (odds ratio (OR) = 4.342, 95% CI 1.764–10.687, *p* = 0.001) in patients who were not given lobaplatin (Wu et al., 2018).

Several studies have explored the association between platinum and *BRCA* mutant subtype prognosis. According to the WSG-ADAPT TN randomized phase II trial, neoadjuvant nab-paclitaxel plus carboplatin indicated an excellent pCR rate of 64% in *BRCA1/2*-mutated cases versus 34.5% in all others mutations (OR = 3.41, 95% CI: 1.11–10.50; *p =* 0.03) supporting the de-escalation strategy in *BRCA1/2* mutations in early TNBC patients (Richters et al., 2021). A study conducted in a neoadjuvant setting with small sample size, involving 12 *BRCA1* mutation carriers, four cycles of chemotherapy with single-agent cisplatin at 75 mg/m^2^ every 21 days yielded a pCR rate of 80% (Byrski et al., 2010). When the same regimen was studied as NAC in 28 patients with TNBC (including 12 *BRCA1* mutation carriers), a pCR rate of 22% was reported (Silver et al., 2010). These two small phase II clinical trials suggest that triple-negative, particularly *BRCA1*-mutant tumors, are more susceptible to DNA-damaging agents such as cisplatin. A retrospective evaluation of 12 patients with BC and *BRCA1* gene mutations also revealed that Pt-based NAC was highly effective in BC patients with *BRCA1* gene mutations (Sæther et al., 2018). Additionally, NeoSTOP trial showed a higher pCR (76 vs. 49%, OR = 3.35, 95% CI: 0.99–11.37; *p =* 0.052) in patients with *BRCA1/2* mutation than the non-*BRCA1/2* mutated patients (Sharma et al., 2021). However, in the secondary analysis of the GeparSixto randomized clinical trial, it was found that patients without *BRCA1/2* mutation showed a higher pCR with the carboplatin (55 vs. 37%, OR = 2.14; 95% CI, 1.28–3.58; *p =* 0.004) compared to the non-carboplatin arm, whereas those with *BRCA1/2* mutation did not significantly improve the pCR with the addition of carboplatin (Hahnen et al., 2017). The secondary analysis of the randomized phase III BrighTNess trial, concurred the benefit of carboplatin across all molecular subtypes of TNBC (Filho et al., 2021). Thus, neoadjuvant therapy improves the pCR rates in patients with TNBC, consequently enhancing the survival benefits and the quality of life (Cortazar et al., 2014).

Clinical Guidelines and consensus conferences provide differing viewpoint regarding the use of Pt- and taxane-based regimens for TNBC. The National Comprehensive Cancer Network (NCCN) Breast Cancer Guidelines (National Cancer Comprehen, 2021), version 5.2021 does not recommend the routine usage of Pt agents as part of NAC in TNBC for a majority of the patients (including *BRCA* mutation carriers). while the adjuvant treatment with Pt agents is discouraged. Also, it is suggested that there is a paucity of data regarding the optimum combination of taxanes and/or ideal chemotherapy regimen in situations where a Pt agent has to be included in an anthracycline-based regimen. However, the guideline suggests the utility of Pt agents in NAC setting only in specific scenarios where local control is imperative.

The European Society of Medical Oncology (ESMO) 2019 clinical practice guidelines for early breast cancer recommend a sequential anthracycline/taxane-based regimen as the standard for the majority of patients. In selected lower-risk patients, four cycles of anthracycline- or taxane-based chemotherapy or cyclophosphamide/methotrexate/5-fluorouracil (CMF) may be used. The addition of a Pt compound may be considered in triple-negative tumors and/or in patients with deleterious *BRCA1/2* mutations (Cardoso et al., 2019).

The panelists at St. Gallen International Consensus Conference for the primary therapy of early breast cancer 2019 recommended against the routine inclusion of Pt-based chemotherapy in women already slated to receive alkylator-, taxane-, and anthracycline-based regimens. However, they favored the inclusion of Pt-based chemotherapy among women with known, deleterious germline *BRCA1/2* mutations, though the evidence supporting this is inadequate. Further, they endorsed dose-dense (accelerated schedules of anthracycline- and alkylator-based therapy, followed sequentially by dose-dense or weekly taxane) treatment as a preferred approach for anthracycline- and taxane-based NAC and adjuvant chemotherapy regimens (Burstein et al., 2019).

The latest guidelines from the American Society of Clinical Oncology (ASCO) suggests that TNBC patients with node-positive and/or at least T1c disease should be given an anthracycline- and taxane-containing regimen while those with either cT1a or cT1bN0 TNBC must avoid NAC. Carboplatin can be added to the NAC regimen for treating TNBC patients to increase the likelihood of pCR. In premenopausal women with hormone-receptor-positive/HER2-negative early-stage BC, endocrine therapy in neoadjuvant setting should be avoided with exception to clinical trials (Korde et al., 2021).

## Progress of Adjuvant Research on Pt Derivatives in Combination With Taxanes

TNBC is associated with a higher risk of recurrence within 3 years, increased risk of distant metastases and brain metastases with rapid progression from distant recurrence to death, as well as absence of known therapeutic targets. It is therefore crucial to optimize the early-stage chemotherapy for such patients (Dent et al., 2007). In daily practice, usual chemotherapy for adjuvant treatment of TNBC includes anthracycline, and taxane-containing regimens, whereas the dose-dense chemotherapy approach is still debated (Joensuu and Gligorov, 2012). The efficacy of Pt added to taxane in the adjuvant setting is still being explored, nevertheless, few recent studies in this therapeutic area are summarized in Table 2.

In a single-arm study, a high pCR rate (65%) was seen in patients with TNBC (n = 74) treated with cisplatin 30 mg/m^2^, epirubicin 50 mg/m^2^, and paclitaxel 120 mg/m^2^ weekly for 8 weeks with granulocyte colony-stimulating factor (G-CSF) on days 3–5. Patients who attained a pCR had a 3-years DFS rate of 97% and a 5-year DFS rate 90% (Frasci et al., 2009).

In a phase II trial, a subset of randomly assigned chemotherapy-naïve patients with TNBC after surgery received six cycles of taxane and platinum (TP) regimen (docetaxel: 75 mg/m^2^ or paclitaxel 175 mg/m^2^; carboplatin AUC = 5, day 1) or epirubicin, cyclophosphamide, and taxane (EC-T) regimen (4 cycles of epirubicin: 90 mg/m^2^; cyclophosphamide: 600 mg/m^2^, day 1 accompanied with four cycles of docetaxel 75 mg/m^2^ or paclitaxel 175 mg/m^2^, day 1) (Du et al., 2020). Both regimens were repeated every 3 weeks. The above study indicated non-inferiority of carboplatin plus taxanes to epirubicin plus cyclophosphamide followed by taxanes (TP vs. EC-T, 5-year DFS = 84.4 vs. 85.8%; absolute difference: 1.4%, 95% CI −5.3–8.1%; *p =* 0.034) as adjuvant chemotherapy for early TNBC (Du et al., 2020).

In 2020, PATTERN (Yu et al., 2020) study reported that when six cycles of paclitaxel with carboplatin were compared with a standard-dose regimen of three cycles of cyclophosphamide, epirubicin, and fluorouracil followed by three cycles of docetaxel (CEF-T), the DFS after a follow-up of 62 months was found to be higher (5-year DFS, 86.5 vs. 80.3%, hazard ratio [HR] = 0.65; 95% CI, 0.44–0.96; *p =* 0.03) in the paclitaxel with carboplatin group versus CEF-T group.

## Future Perspectives and Implications

In light of the current advancement towards treating TNBC, the combination of Pt to neoadjuvant taxane-based chemotherapy results in favorable outcomes, with a majority of the studies pointing toward a higher pCR. Evidence favor carboplatin with docetaxel in the NAC regimen, which yields an excellent pCR in patients with *BRCA*-associated and wild-type TNBC (Sharma et al., 2017).

Historically, *BRCA1/2* mutations were regarded as an accessible biomarker for predicting longer PFS and clinical outcomes with carboplatin in comparison with docetaxel under adjuvant settings (Tutt et al., 2018). However, results from the randomized, phase III, TNT trial revealed that one-third of the TNBC patients with *BRCA1/2* mutation were non-responders to the Pt therapy. This may arise due to the homologous recombinant repair (*HRR*) gene defect that is retained in *BRCA1/2* mutation carriers that forms a hard epigenetic *BRCA*ness (Tutt et al., 2018).

GeparSixto (von Minckwitz et al., 2014) and BrighTNess (Loibl et al., 2018) trials reported similar pCR benefits in *BRCA* mutated cohorts in patients receiving Pt-based chemotherapy compared to the non Pt containing arms. The secondary analysis of the BrighTNess phase III randomized clinical trial also showed that the addition of carboplatin to standard NAC can yield pCR benefits across all the molecular subtypes (Filho et al., 2021). Compared to the other BC subtypes, 11–31% of women with TNBC are found to have germline *BRCA* mutations (Cocco et al., 2020). Although these studies show that the Pt agents under neoadjuvant setting may significantly improve the pCR in TNBC patients regardless of the gBRCA1/2 mutation status, their effectiveness remains debatable to date. This critique can be attributed to the fact that the supporting evidence was derived from a post hoc exploratory analysis with a small number of *BRCA*-mutated patients. Moreover, no clear recommendations are provided by current guidelines on the usage of Pt in neoadjuvant settings for TNBC.

The poly ADP-ribose polymerase inhibitors have also shown huge potential with promising clinical efficacy and lower toxicity profiles when given in monotherapy in TNBC with *BRCA1/2* mutations (Guney Eskiler et al., 2018). These effects are due to HRR deficiency that results in faulty DNA repair mechanisms. In phase II BROCADE (Han et al., 2018) trial, there was a statistically significant increase in ORR from 61.3 to 77.8% when veliparib was added to carboplatin with paclitaxel regimen versus carboplatin with paclitaxel alone. An increase in median PFS (14.1 vs. 12.3 months, HR = 0.789; 95% CI 0.536–1.162; *p =* 0.227) and OS (28.3 and 25.9 months, HR = 0.750; 95% CI 0.503–1.117; *p =* 0.156) was also observed when veliparib was added to the Pt-based taxane chemotherapy in metastatic BC. (Han et al., 2018)

On the other hand, biomarkers such as tumor-infiltrating lymphocytes (TILs) in TNBC are linked to a higher mutation rate and pCR with NAC and an improved survival outcome with adjuvant therapy (de Boo et al., 2020). Another common biomarker that is expressed in 20% of TNBC is PD-L1 (Cocco et al., 2020). Tumor mutational burden (TMB) being a good marker of tumor antigenicity has a high prevalence in TNBC. Moreover, PI3K, AKT, and mammalian target of rapamycin (mTOR) pathway alterations also occur in approximately 35% of TNBC (Cocco et al., 2020).

Although conducting studies in the adjuvant setting with Pt-based treatment is fairly feasible, the complexity in obtaining enriched tumor samples for research purposes is an enormous challenge (Agrawal and Mayer, 2014). Pt resistance due to prior exposure to Pt agents in preoperative settings can lead to increased toxicity during adjuvant therapy and is possibly the cause of inadequate and sparse studies reinforcing Pt-based adjuvant therapy (Agrawal and Mayer, 2014). In addition, a relatively higher survival benefit that is offered by the conventional adjuvant therapies might be associated with the modicum of studies evaluating Pt- and taxane-based combination therapy in the adjuvant setting. Also, the utility of Pt-based adjuvant therapy is highly controversial as there are insufficient trials assessing the DFS and the OS in such regimens. A meta-analysis revealed higher DFS (HR = 0.73, 95% CI 0.59–0.91, *p =* 0.005) as well as OS improvement (HR = 0.69, 95% CI 0.56–0.85) from adjuvant addition of capecitabine to four cycles of epirubicin and cyclophosphamide. in patients with early TNBC, treated with neoadjuvant carboplatin and docetaxel chemotherapy and without pCR data (Li et al., 2020). The accumulating toxicities of Pt agents can present barriers for the long-term use of these agents. Previous *in vivo* studies have also suggested that cells resistant to platinum often become sensitive to taxanes and vice-versa. As a result, combination of these two drugs is feasible for treatment of early TNBC. A phase II safety and efficacy study in preoperative weekly cisplatin-epirubicin-paclitaxel support in operable TNBC showed high 3- and 5-year DFS rates of 97 and 90% respectively, in contrast to the DFS rates of 61 and 56%, respectively, in those with residual disease after NAC. The numbers of patients with T2 and T3 tumors were same, however the pCR rate was significantly higher in the T2 tumors (74 versus 51%) (Frasci et al., 2009). Thus, the findings from the majority of the available studies suggest that the regimens containing both taxane plus Pt agents might be an effective alternative in adjuvant settings for patients with operable TNBC (Foulkes et al., 2010; Liedtke and Rody, 2017; Yu et al., 2020).

Many studies are underway that are directed towards evaluating the advantages of adding Pt to various adjuvant and NAC regimen for treating TNBC (Table 3). CALGB40603 (Sikov et al., 2015) is one such ongoing trial that is set to assess the long-term benefits of adding weekly paclitaxel to carboplatin in neoadjuvant setting. So far, the results are promising with a high pCR, but the survival outcomes such as RFS and OS are awaited (Sikov et al., 2015).

**TABLE 3:** Summary of ongoing and recently reported clinical trials with Pt derivatives and taxane combinations in triple-negative breast cancer.

phase	NCT	Study population	Setting	Stage	Experimental arm	Control arm	Primary endpoint
II	NCT01042379	TNBC	Neoadjuvant	II–III	Veli + Cb → standard NACT	Standard NACT	pCR
III	NCT02032277 (BrighTNess)	TNBC	Neoadjuvant	II–III	Veli + Cb + P → AC	Placebo + Cb + P → AC	pCR
II/III	NCT03150576 (PARTNER)	TNBC and/or gBRCA mutated BC	Neoadjuvant	II–III	Ola + Cb + P → AC/EC	P + Cb → AC/EC	Safety, pCR
II	NCT03639948 (NeoPACT)	TNBC	Neoadjuvant	I–III	Cb + T + pembro	NA	pCR
III	NCT02620280 (NeoTRIPaPDL1)	High-risk TNBC	Neoadjuvant	II–III	Cb + nab-P + atezo → AC/EC/FEC	Cb + nab-P → AC/EC/FEC	EFS
III	NCT03036488 (Keynote-522)	TNBC	Neoadjuvant	II–III	Cb + P + pembro → AC/EC + pembro	Cb + P + placebo → AC/EC + placebo	pCR, EFS
III	NCT03281954 (NSABP B-59)	TNBC	Neoadjuvant	II–III	P + Cb + atezo → atezo + AC/EC	P + Cb + placebo → placebo + AC/EC	pCR, EFS
II	NCT03872505 (PANDoRA)	TNBC	Neoadjuvant	II–III	Durva + Cb + P + radiation	Durva + Cb + P	pCR
II	NCT03650738	TNBC	Neoadjuvant	II–III	Apatinib + nab-P + Cb	NA	pCR, safety
II	NCT03193853	TNBC	Neoadjuvant	IV	Tak-228 + Tak-117 → cis + nab-P	NA	ORR
II/III	NCT02221999	TNBC and Hormone-receptor-positive	Neoadjuvant	III-IV	P + cis + leuprolide/goserelin versus P + cis + letrozole	P + cis	pCR
II	NCT04537286	TNBC	Neoadjuvant	IV	Nab-P + cis + carilizumab	NA	PFS, safety
II	NCT04159142	TNBC	Neoadjuvant	III	Nab-P + Cb	Nab-P + capecitabine	PFS
II	NCT03121352	TNBC	Neoadjuvant	IV	Cb + nab-P + pembro	NA	ORR
II	NCT04083963 (BRE-01)	TNBC	Neoadjuvant	I-IV	P + Cb → AC/EC	NA	pCR
II	NCT02876107	TNBC	Neoadjuvant	I-III	P + Cb + panitumumab	P + Cb	pCR
II	NCT02124902	TNBC	Neoadjuvant	II–III	T + Cb	T + Cb	pCR
IV	NCT04136782	TNBC	Neoadjuvant	II-III	Nab-P + Cb	E + T	pCR
II	NCT02547987 (CADENCE)	TNBC	Neoadjuvant	II-III	T + Cb	NA	pCR
II	NCT01525966	TNBC	Adjuvant	II–III	Cb and nab-P	NA	pCR
III	NCT02455141 (TCTN)	TNBC	Adjuvant		EC → P or T	EC → P or T + Cb	DFS
III	NCT03876886	TNBC	Adjuvant	II-III	AC + P	P + Cb	DFS
III	NCT02441933 (PEARLY Trial)	TNBC	Adjuvant/Neoadjuvant	II-III	AC → P or T + Cb	AC→ P or T	DFS

A, doxorubicin; Atezo, atezolizumab; C, cyclophosphamide; Cb, carboplatin; Cis, cisplatin; DFS, Disease-free survival; Durva, durvalumab; EFS, event-free survival; E, epirubicin; F,5-fluorouracil; NA, not available’ nab-P, albumin paclitaxel (weekly cycle if not specially noted); ORR, objective response rate; Ola, olaparib, P, paclitaxel (weekly cycle if not specially noted), PCR, pathological complete response, Pembro, pembrolizumab; RFS, Relapse-free survival; T, docetaxel; TNBC, triple negative breast cancer; Veli, veliparib.

## Conclusion

The use of Pt- and taxane-based chemotherapy in the neoadjuvant and adjuvant setting has tremendous potential to improve survival of patients with early TNBC by achieving a high pCR. TNBC in general provides a therapeutic challenge that can be tackled by identifying new therapeutic sub-targets and a specific subgroup that can benefit from a Pt- and taxane-based chemotherapy. Results from ongoing trials are expected to further validate the clinical benefits of this combination, especially in patients with early-stage or operable TNBC.

## Glossary

AUC: area under curve
BC: breast cancer
CI: confidence-interval
CVAP: cyclophosphamide/vincristine/doxorubicin/prednisolone
CYP: cytochrome P-450
DDFS: distant disease-free survival
DFS: disease-free survival
EFS: event-free survival
ER: estrogen receptor
G-CSF: granulocyte colony-stimulating factor
HER2: human epidermal growth factor receptor-2
*HRR*: homologous recombinant repair
mTOR: mammalian target of rapamycin
mDFS: median disease-free survival
mOS: median overall survival
miRNAs: MicroRNAs
mRNAs: messenger RNAs
MRP‐1: multidrug resistance protein-1
MDR: multidrug resistance
NAC: neoadjuvant chemotherapy
OR: odds ratio
ORR: objective response rates
OS: overall survival
PFS: progression-free survival
Pt: platinum
pCR: pathological complete response
PR: progesterone receptor
RFS: relapse-free survival
5-FU: 5‐fluorouracil
TNBC: triple-negative breast cancer
TILs: tumor-infiltrating lymphocytes
TMB: tumor mutational burden
