# Supplementary figures and images for: Platinum and Taxane Based Adjuvant and Neoadjuvant Chemotherapy in Early Triple-Negative Breast Cancer: A Narrative Review
Source: Front Pharmacol. 2021 Dec 6;12:770663. doi: 10.3389/fphar.2021.770663 (PMC8685522; doi:10.3389/fphar.2021.770663)

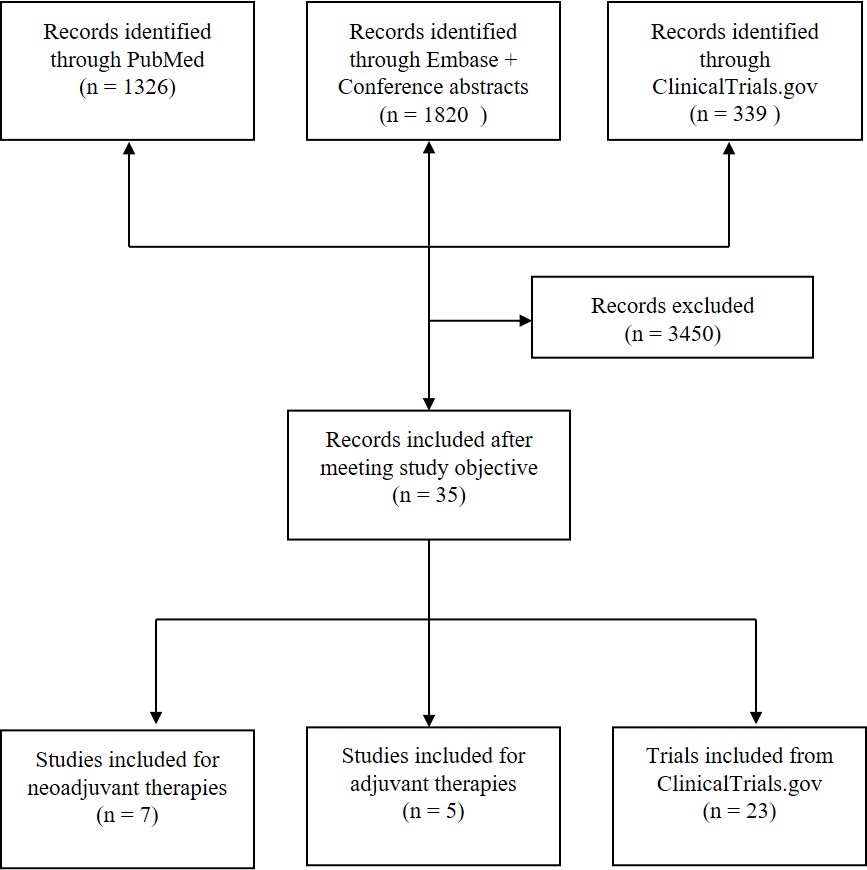

Supplement: Supplementary file 1 [file Image1.jpg]
